# Histone Modification Is Involved in Okadaic Acid (OA) Induced DNA Damage Response and G_2_-M Transition Arrest in Maize

**DOI:** 10.1371/journal.pone.0155852

**Published:** 2016-05-19

**Authors:** Hao Zhang, Pu Wang, Haoli Hou, Huan Wen, Hong Zhou, Fei Gao, Jinping Wu, Zhengming Qiu, Lijia Li

**Affiliations:** 1 State Key Laboratory of Hybrid Rice, College of Life Sciences, Wuhan University, Wuhan, China; 2 Industrial Crops Institute of Hubei Academy of Agricultural Sciences, Hongshan District, Wuhan City, Hubei Province, China; St. Georges University of London, UNITED KINGDOM

## Abstract

Histone modifications are involved in regulation of chromatin structure. To investigate the relationship between chromatin modification and cell cycle regulation during plant cell proliferation, Okadaic acid (OA), a specific inhibitor of serine/threonine protein phosphatase, was applied in this study. The results showed that OA caused the cell cycle arrest at preprophase, leading to seedling growth inhibition. Western blotting assay revealed that the spatial distribution of phosphorylation of Ser10 histone H3 tails (H3S10ph) signals was altered under OA treatment. Reactive oxygen species (ROS) was found to be at higher levels and TdT-mediated dUTP nick end labeling (TUNEL) assay displayed DNA breaks happened at the chromatin after treatment with OA, companied with an increase in the acetylation of histone H4 at lysine 5 (H4K5ac) level. From these observations, we speculated that the alteration of the spatial distribution of H3S10ph and the level of H4K5ac was involved in the procedure that OA induced DNA breaks and G2-M arrested by the accumulation of ROS, and that the histone H3S10ph and H4K5ac might facilitate DNA repair by their association with the chromatin decondensation.

## Introduction

Recent data shows that epigenetic marks including DNA and histone modification are involved in chromatin DNA replication and genetic transmission during the cell cycle [1]. Study of dynamics of epigenetic marks through cell cycle will be helpful for understanding mechanisms and means that ensure the correct information transmission in cell division. During last few years numerous research focusing on the role of histone modification have been reported [2, 3]. N-terminus tails of histones could be catalyzed by various enzymes, leading to different modifications, such as acetylation, methylation and phosphorylation [4]. These histone modifications alter chromatin structure and accessibility of transcription complexe, and then regulate gene expression [5]. For instance, hyperacetylated histone is generally located on transcriptional active chromatin region and deacetylated histone always appears on repressive chromatin regions [6, 7]. It has been shown that chromosomal distribution of individual histone modifications differs along the cell cycle, suggesting that the function of these modifications is complicated and dynamic during the cell cycle [8, 9].

Chromatin decondensation of the eukaryotic cell is necessary for replication, transcription and repair [8], whereas chromatin condensation is extremely vital during mitosis that insures the genetic material distributed to two daughter-cells equally and accurately. Phosphorylation of Ser10 histone H3 tails (H3S10ph) is a key histone modification in mitosis, existing at the pericentromeric zones from preprophase to telophase, which is conserved among eukaryotes, including animals and plants [10, 11]. It is reported that H3S10ph is involved in chromatin decondensation and gene expression [8, 12]. Besides, recent studies indicate that H3S10ph also correlates with chromosome condensation in mitosis [4]. It remains to be explored that how the phosphorylation of H3S10 affects the structural change of chromatin.

The present study has established the role of reactive oxygen species (ROS) in the cell cycle arrest [13, 14]. ROS generated in cells is known to cause serious damage to DNA. Cells trigger DNA damage checkpoint response when sense DNA breaks and arrest cell-cycle progression until DNA break is repaired [15]. Histone acetylation has been showed to be involved in DNA repair [16]. Histone acetylation can mediate decondensation of the nucleosome structure, which makes chromatin more accessible to nuclear protein complexes [17]. Decondensed chromatin is necessary for the repair of DNA breaks, and cells with DNA breaks always keep high levels of acetylation, suggesting that the acetylation of histone creates a favorable chromatin environment for DNA repair [18].

Almost all types of protein phosphatases known in yeasts and mammals have also been identified in various higher plants [19]. Serine/threonine-specific protein phosphatases family are classified into the PPP and PPM subfamilies. The PPP subfamily includes the phosphatase PP1, PP2A, PP2B, and PP3–PP7 and the PPM family includes PP2C [20, 21]. It was revealed that the use of a high concentration of endothall (ET), a PP1 and PP2A inhibitor, increased the frequency of hypercondensed early and late prophase chromosomes that could not enter metaphase [22].

Okadaic acid (OA) is a powerful inhibitor of protein serine/threonine phosphatases-1 and -2A (PP1 and PP2A) [23]. It is widely adopted that treating plants such as maize to study the effect of PP1 and PP2A activities on signal transmitting and gene activation [24, 25]. In addition, OA has also been widely used to alter the state of histone phosphorylation in cell cycle progression and is a very good agent for investigating the relationship between chromatin modification and cell cycle progression [26, 27]. After OA treatment, the chromosomes in *Vicia faba* cells became highly condensed in the arrested mitotic cells, but the chromosomes became scattered under prolonged treatment [28]. In this study, OA was applied for studying the relationship between chromatin change and the cell cycle progression during the plant growth in maize. Our results suggested that OA could cause preprophase arrest via ROS accumulation, with DNA damage and elevation of histone H4K5ac, which was related to chromatin condensation.

## Materials and Methods

### Plant materials

Seedlings of maize (*Zea mays* L. inbred line Huayu 5, provided by Hubei Provincial Seed Group Co.,LTD) were sterilized and germinated, and then grown with water culture method for 72h at 25°C, no light condition and 70% relative humidity [29]. The 100 nM OA (S1786, Beyotime) was applied to stimulate the root of maize seedlings, based on the working concentration in maize reported by Sheen (1993) [25]. To initiate planned treatment, 3d-old seedlings were distributed into two groups, which were treated with distilled water and 100 nM OA respectively. After 24 h, 48 h and 72 h, leaves from different conditions were collected and processed for different assays.

### Antibodies

The following antibodies were used for immunostaining and western blotting:anti-H3K9ac (07–352), anti-H3ac (06–599), anti-H3S10ph (06–570), fluorescein-conjugated goat anti-rabbit IgG (16–237) and anti-H3K4me2 (07–030) were produced by Millipore (Millipore, Billerica, MA, USA). And anti-H4K5ac (ab51997), anti-H3 (ab1791), anti-γH2AX (ab2893) and AP-conjugated goat anti-rabbit IgG (A4187) were purchased from Abcam (Abcam, Cambridge, MA, USA).

### Growth analysis

Images of seedlings were captured to measure the length of maize leaves and roots. The length of each sample was quantified using the software ImageJ (NIH, Bethesda, MD, USA).

### Flow Cytometry

Nuclei were isolated based on the described method [30]. The samples were chopped rapidly with nucleus isolation buffer [10 mM MgSO4, 50 mM KCl, 5 mM Hepes, 1 mg /ml dithiothreitol (Sigma, St. Louis, MO) and 0.2% Triton X-100] and filtered through a 33 μm nylon mesh. The nuclei were fixed in 4% paraformaldehyde for 30 min at 4°C, then were precipitated (200 g, 10 min, 4°C), resuspended in the isolation buffer and stained with Propidium Iodide (PI). The cell-cycle profile was determined with a FACSCalibur flow cytometer (Becton Dickinson, San Jose, CA, USA) equipped with an argon-ion laser, using the 488 nm laser line for excitation. The results of Flow Cytometry were quantified using the software Flowjo 7.6.

### Mitosis phase counting

The edge of leaf samples was collected and fixed in Carnoy's Fluid (a solution of ethanol and gladual acetic acid in a 3:1 volume ratio) at 4°C for 30 min. Afterwards, leaf edges were washed several times with 70% ethanol, followed by enzymolysis in a mixture of 2% pectinase and 4% cellulase at 37°C for 2 h. The digested leaf edge was squashed in a drop of 1×PBS on a slide below a coverslip. After removal of the coverslip, all slides were stained with DAPI (Sigma, St. Louis, MO, USA), mounted with Vectashield (Vector labs, Burlingame, CA, USA) and examined under a fluorescence microscope (Olympus BX-60). Images were captured with a CCD monochrome camera Sensys 1401E. Microscope settings and camera detector exposure times of each channel were kept constant respectively. More than 1000 nuclei were measured for each treatment group.

### Immunostaining

Nucleus isolation and immunostaining were performed according to the described method [31]. Nuclei were spread on a slide, incubated with 3% bovine serum albumin at 37°C for 1h, followed by overnight incubation at 4°C with primary antibody and 2h incubation at 37°C with secondary antibody. Secondary antibodies were conjugated with FITC. All slides were stained with DAPI, mounted with Vectashield and examined under an Olympus BX-60 fluorescence microscope with filter blocks for DAPI and fluorescein. Images captured with a CCD monochrome camera Sensys 1401E were pseudo-colored and merged using the software MetaMorphH 4.6.3. Microscope settings and camera detector exposure time of each channel were kept constant respectively. More than 500 nuclei were detected for each treatment group. For both control and treated groups, three independent immunostaining experiments were performed with each antibody.

### TUNEL assay

TUNEL staining was performed using the Deadend™ Fluorometric TUNEL System Kit (G3250). Nucleus isolation was performed according to the method described previously [31]. After centrifugation under 300 g for 10 min at 4°C, nuclei were resuspended in the isolation buffer and then fixed in 4% paraformaldehyde (pH 7.3) for 20 min. Next, the mixture was centrifuged under 300 g for 10 min at 4°C. Nuclei were resuspended in 80 μl equilibration buffer, followed by incubation for 5 min. After centrifugation under 300 g for 10 min, terminal deoxynucleotidyl transferase (TdT) reaction buffer (containing 45 μl incubation buffer, 5 μl of a fixed concentration buffer of dATP, dGTP, dCTP and fluorescein-12-dUTP, and 1μl TdT) were applied to serial sections for 1 h at 60°C, then the stop buffer (EDTA) was added. Nuclei were spread on a slide and all slides were stained with DAPI (Sigma, St. Louis, MO, USA), mounted with Vectashield (Vector labs, Burlingame, CA, USA) and examined under an Olympus BX-60 fluorescence microscope with filter blocks for DAPI and fluorescein. Images captured with a CCD monochrome camera Sensys 1401E were pseudo-colored and merged using the software MetaMorph 4.6.3 (Universal Imaging Corp., Downingtown, PA, USA).

### Quantitative real-time PCR

The first-strand cDNA was synthesized from total RNA isolated from leaf samples with Trizol reagent (Invitrogen, Carlsbad, CA, USA) by using Revert Aid First Strand cDNA Synthesis Kit (Fermentas, Canada). Quantitative real-time PCR was carried out using SYBR® Green Real-time PCR Master Mix (TOYOBO, Japan) in a StepOne Plus real-time PCR system (Applied Biosystems, Carlsbad, USA) with the following cycling conditions: 94°C for 2 min, followed by 40 amplification cycles at 94°C for 5 s, 56°C for 15 s, and 72°C for 20 s. All primers used for quantitative real-time PCR of topoisomerase genes are listed in Table 1. Fluorescence data were acquired at the 72°C step and during the melting-curve program. To ensure the amplification of a single PCR product for each gene, preliminary experiments were conducted. Relative expression levels were normalized with the maize β-actin gene [32]. Quantitative PCR primers were designed to amplify ca. 200 base pair (bp) fragments. RT-PCR was repeated three times for each sample from three independent experiments.

**Table 1:** Primers used for real-time PCR of topoisomerase genes.

Genes	RT-PCR Primer Sequences (5’-3’)
*Actin*	GATGATGCGCCAAGAGCTG(F) GCCTCATCACCTACGTAGGCAT (R)
*DNA topoisomerase I*	CCGTCATCCAGTCCAAACCA(F) TCCTTAACCCAATCGGCACC (R)
*putative DNA Topoisomerase I*	TGGAAAAGGACAATGGCTTGC(F) TGCTGGACTGGCCTGATTC (R)
*DNA topoisomerase 2-like*	AGTAGTCGCAAAGCGTGGAG(F) ATCCAAGCCAAGCAGATTTGAC(R)
*DNA topoisomerase 2-binding protein 1*	TTCATTCTCCCGCCAGTTCC(F) CCGCGACAGAAACACACAAG (R)
*DNA topoisomerase 6*	TCGCCACATGATTCTACTGGG(F) CACTGCTTCAGGGCTGACTT(R)
*topoisomerase I*	ACCAACAGCGTAGTGTCTCG(F) CCTCTTTGGCTTCCCCTCTG (R)
*PP1*	ACGACAGCGAGAGGAACATC(F) CTTGTCGGAGGAGCGTTCAG (R)

### Western blotting assay

Proteins were extracted by grinding leaf samples in liquid nitrogen and resuspended in extraction baffer (100 mM Tris-HCl pH 7.4, 50 mM NaCl, 5 mM EDTA and 1 mM PMSF). Protein were loaded onto a 12% SDS-PAGE gel and separated by electrophoresis and then transferred to nitrocellulose membranes which were incubated with primary and secondary antibodies step by step [30]. Detection was performed using alkaline phosphatease (AP) conjugated anti-rabbit IgG antibody and chemiluminescence visualization. The mean gray value of the signals of H3S10ph and H4K5ac was measured with ImageJ 1.48 software. Abundance index was calculated as H3S10ph or H4K5Ac band intensity/H3 band intensity. Western blots were repeated three times. Histone H3 was used as equal loading control.

### ROS and related enzyme activity detection assays

Leaves were differently ground in PBS buffer (0.04575 mol/L Na2HPO4, 0.00425 mol/L NaH2PO4, pH 7.8) mixed with quartz sands. Supernatant was collected after centrifugation (8000 g, 20 min) for detection. Supernatant was used for superoxide dismutase (SOD), peroxidase (POD) and catalase (CAT) activity detection. SOD assay was performed according to Wong et al [33]. One unit of SOD was equal to amount which caused a 50% decrease of NBT reduction. POD assay was performed according to Wu et al [34]. Enzymatic activity was determined by catalyzing decomposition of H_2_O_2_ in the presence of guaiacol. One unit of POD was equal to that amount which decreased the absorbance at 470 nm by 0.1 per minute. SOD and POD activity were expressed as U g-1 FW. CAT was assayed according to the reported procedure [35]. The decomposition degree of H_2_O_2_ was detected at 240 nm wavelength. The concentration of the atomic oxygen radical anion (O_2_^-^) was measured according to Li et al [36]. The supernatant was added to a mixture composed of 10 mM hydroxylamine hydrochloride and 0.05 M PBS buffer (pH 7.8). After incubation, 7 mM α-naphthylamine and 17 mM sulfanilamide were added to the mixture. The light absorbance was measured at 530 nm wavelength.

### Statistical analysis

Each data and error bars were calculated from three independent experiments. Data in this manuscript was analysed for significant differences between the treated and control groups by means of t-test. It was considered statistically significant when P < 0.05.

## Results

### Suppression of maize seedling growth and alteration of cell cycle by Okadaic acid

**Fig 1:**
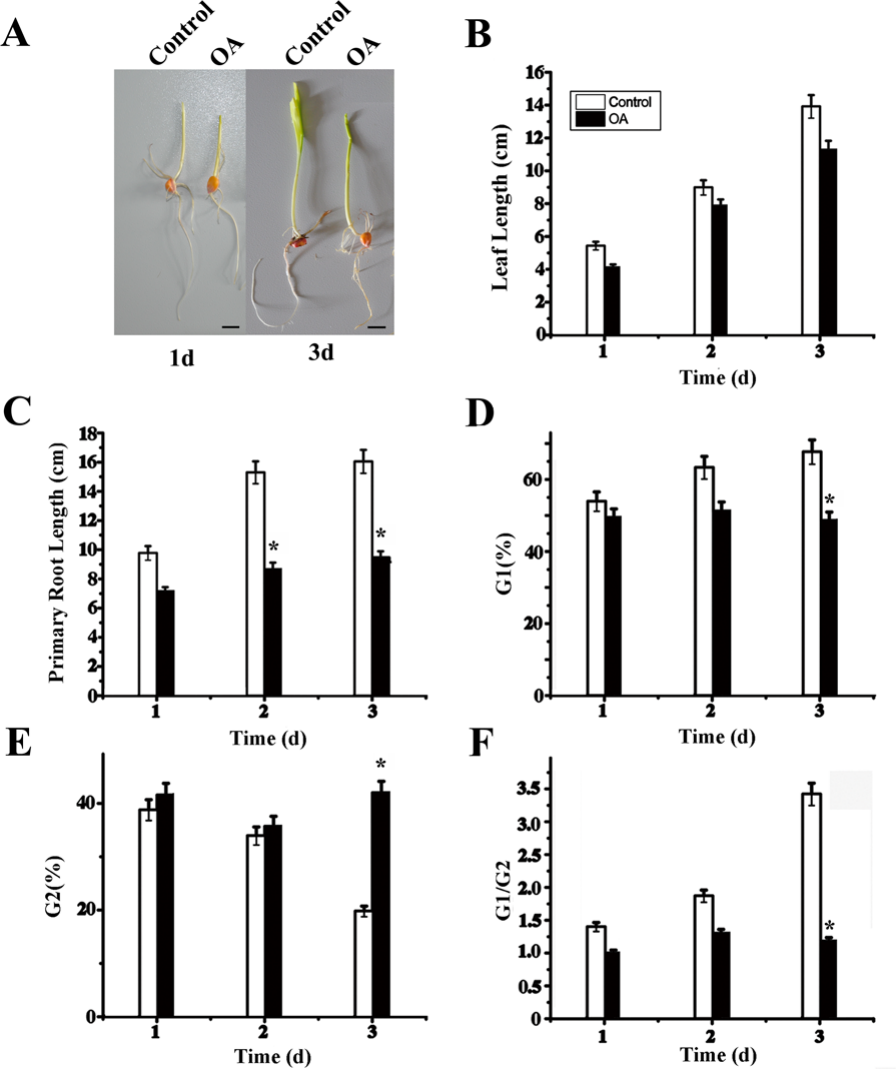
Effects of Okadaic acid on the growth of maize seedlings. (A) Average primary root length with or without OA (n > 100 seedlings). (B) Seedling leaf length after treatment with or without OA (n > 100 seedlings). (C) The percentage of G_1_ phases of leaves cell in the cell cycle analyzed by Flow cytometry under OA treatment. (D) The percentage of G2 phases of leaves cell in the cell cycle analyzed by Flow cytometry under OA treatment. (E) The ratio of G_1_ and G_2_ phases. The standard errors are calculated from three independent measure assays or three flow cytometric assays. Bars = 1cm. *P<0.05, as compared to the control group by t-test.

To investigate the relationship between histone phosphorylation and chromatin condensation during plant cell proliferation, Okadaic acid (OA), a specific inhibitor of serine/threonine protein phosphatase, was applied to treat maize seedlings. First, we valued the growth state of maize seedlings during OA treatment by measuring primary root length and leaf length. The results showed that primary root and leaf length of treated groups was shorter than that in the control group, revealing the suppression of seedling growth caused by Okadaic acid (Fig 1A–1C). Furthermore, cell cycles of both group seedlings were analyzed using Flow Cytometry and Flowjo software. The results showed that the rates of G1 phases were decreased and the rates of G2 phase significantly increased after treatment with OA compared with the control group (Fig 1D and 1E). The inhibition effect of Okadaic acid was increasing significant within the treated period because the G1/G2 ratio of the control group was gradually raised while the seedlings exposed to OA revealed a constant and lower level (Fig 1F). These data suggested that Okadaic acid could arrest of the cell cycle of maize seedlings leading to growth inhibition.

### Effect of Okadaic acid on the phosphorylation of H3S10 in the overall level

**Fig 2:**
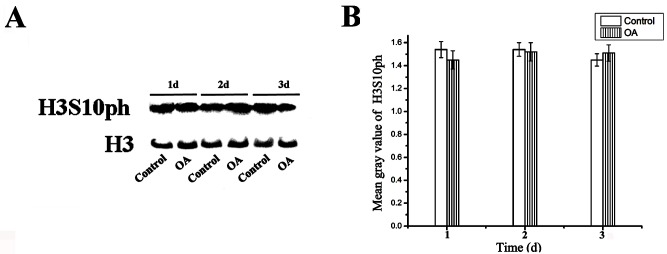
The invariability of the global histone phosphorylation levels after 100nM OA treatment. (A) Western blot assay showed that no obvious alteration of overall H3S10ph signal levels occurred during OA treatment. Histone H3 was applied as an equal loading control. (B) The histogram quantitatively displaying the mean gray values of H3S10ph that were not significantly increased after treatment with OA for 1–3 d. The standard errors are calculated from three independent gray values measure assays.

qPCR analysis of *PP1* was performed showing that OA actually had an effect on the enzymes that it targets (S1 Fig). In addition, we performed western blotting assay with proteins from the control and treated group to investigate the state of histone phosphorylation during the arrest of cell cycle, using specific antibodies against H3S10ph and H3. The result showed that the total H3S10ph levels of the genome were unchanged in both control and treated groups (Fig 2A and 2B).

### Okadaic acid affects spatial distribution of H3S10ph on chromosomes in mitosis

**Fig 3:**
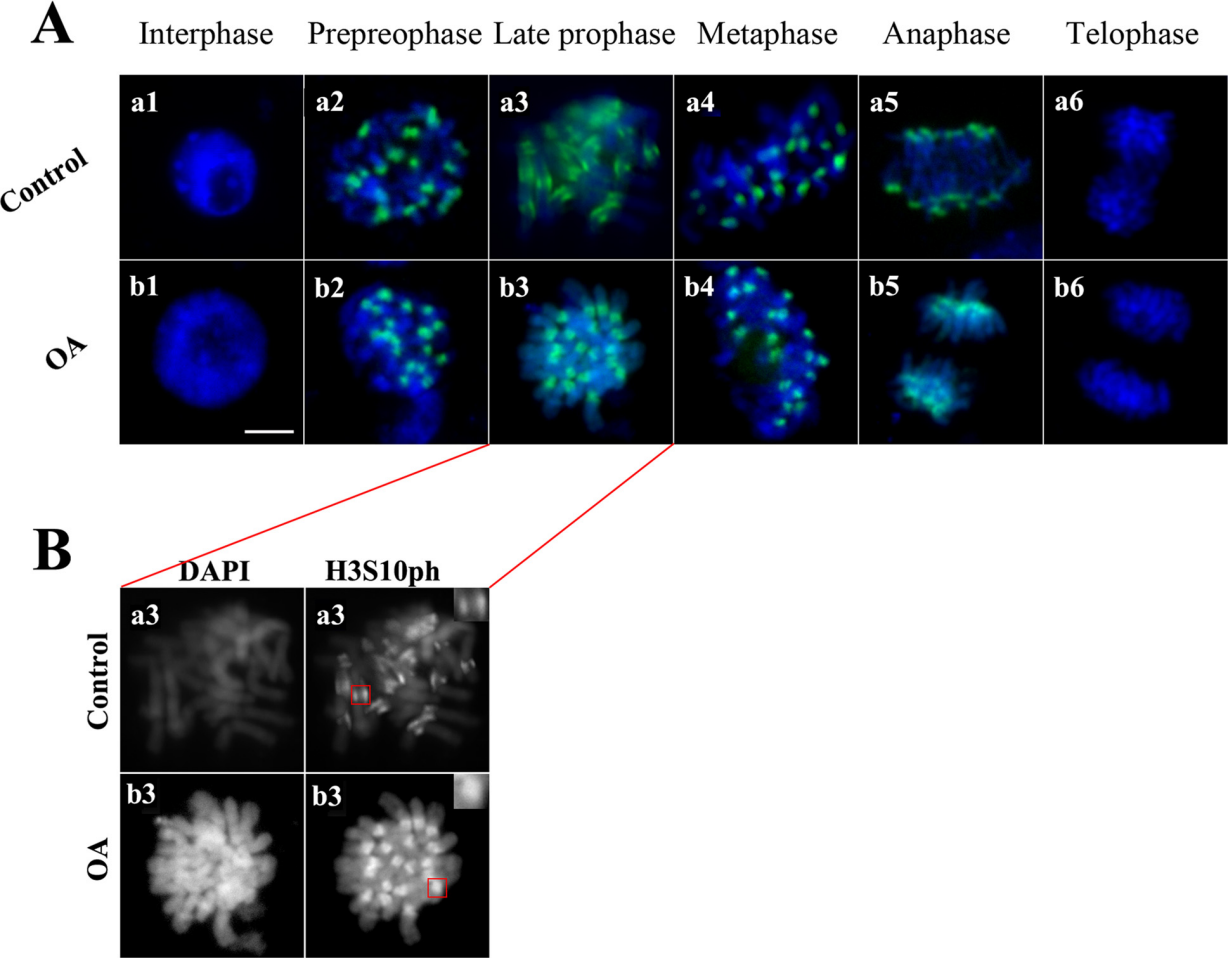
OA affects the spatial location of H3S10ph on chromosomes. (A) Nuclei from leaves of untreated maize seedlings (control) and seedlings treated with 100 nM OA for 72 h (OA) were immunostained with the antibody against H3S10ph and counterstained with DAPI. (B) Morphology analysis of the H3S10ph signal in late prophase.

To further investigate the alteration of H3S10ph on chromosome structures, we detected the distribution of the H3S10ph signals throughout cell cycle following OA treatment by Immunostaining assay. The results showed that H3S10ph signals appeared at preprophase and disappeared in telophase in the control and treated group, consistent with the pervious reported results (Fig 3A). However, interestingly, OA treatment obviously changed the spatial distribution of H3S10ph on chromosomes, as the two regular H3S10ph signals located close to the centromeres turn to the dispersion signal, revealing the way that OA affect phosphorylation of H3S10 (Fig 3A and 3B).

### The cell cycle is arrested at preprophase due to DNA damage

**Fig 4:**
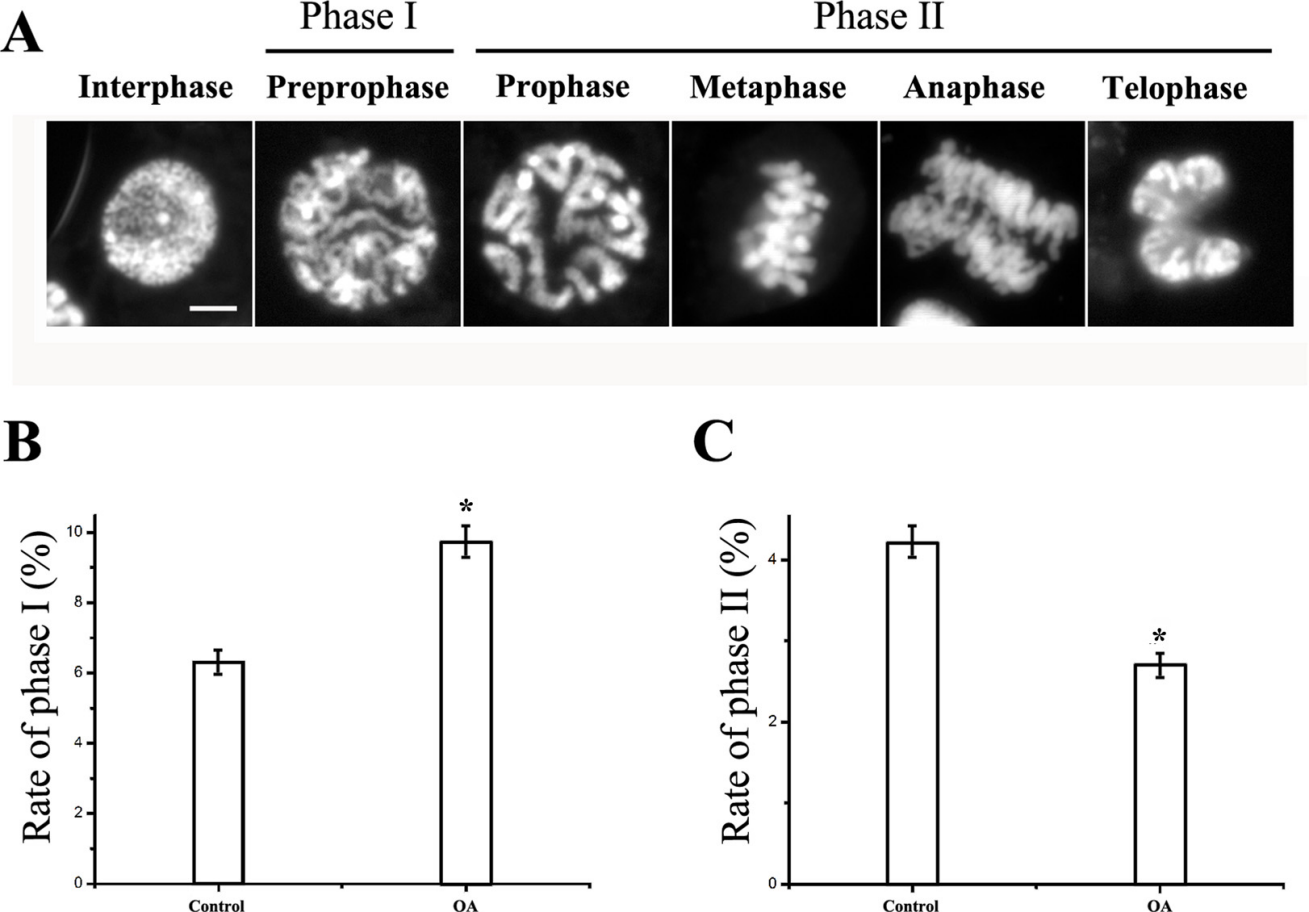
Quantitative analysis of cells in different phases. (A) Morphology of chromatin in different phases of mitosis.The chromatin was visualized by staining with DAPI, mounted with Vectashield and examined under a fluorescence microscope. (B) Quantitative analysis of cells in phase I (preprophase). (C) Quantitative analysis of cells in phase II. More than 300 cells were analyzed for each experiment. Bar = 10 um. *P<0.05, as compared to the control group by t-test.

**Fig 5:**
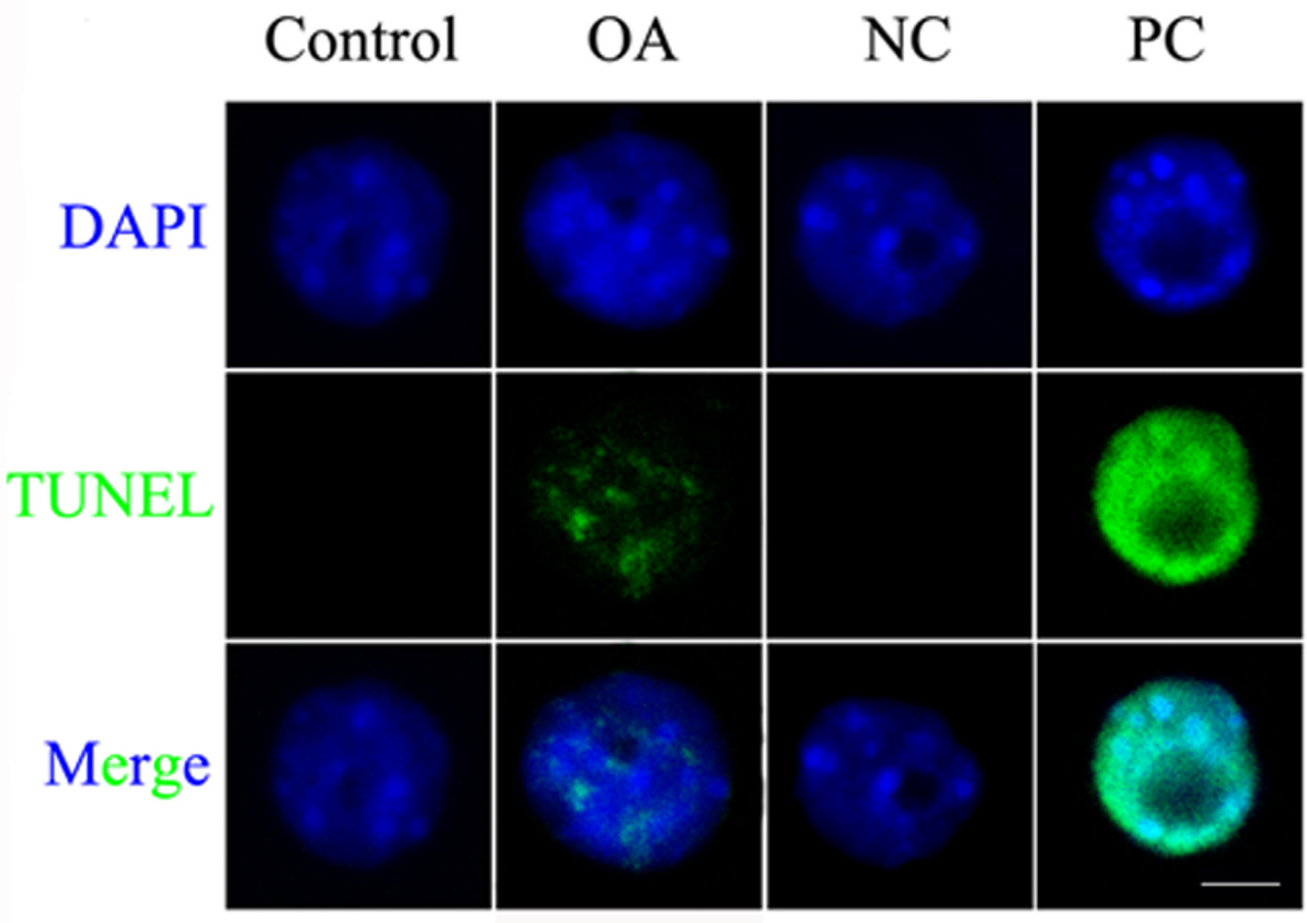
DNA damage response was detected in the maize leaf cells after OA treatment. TUNEL assay showed that OA caused DNA damage. Nuclei in “NC” group were detected without enzyme and nuclei in “PC” group were digested by micrococcal nuclease before detected. “NC” = Negative Control; “PC” = Positive Control. Nuclei were stained with DAPI (blue). More than 300 nuclei were analyzed for each experiment. Bar = 10 μm.

**Fig 6:**
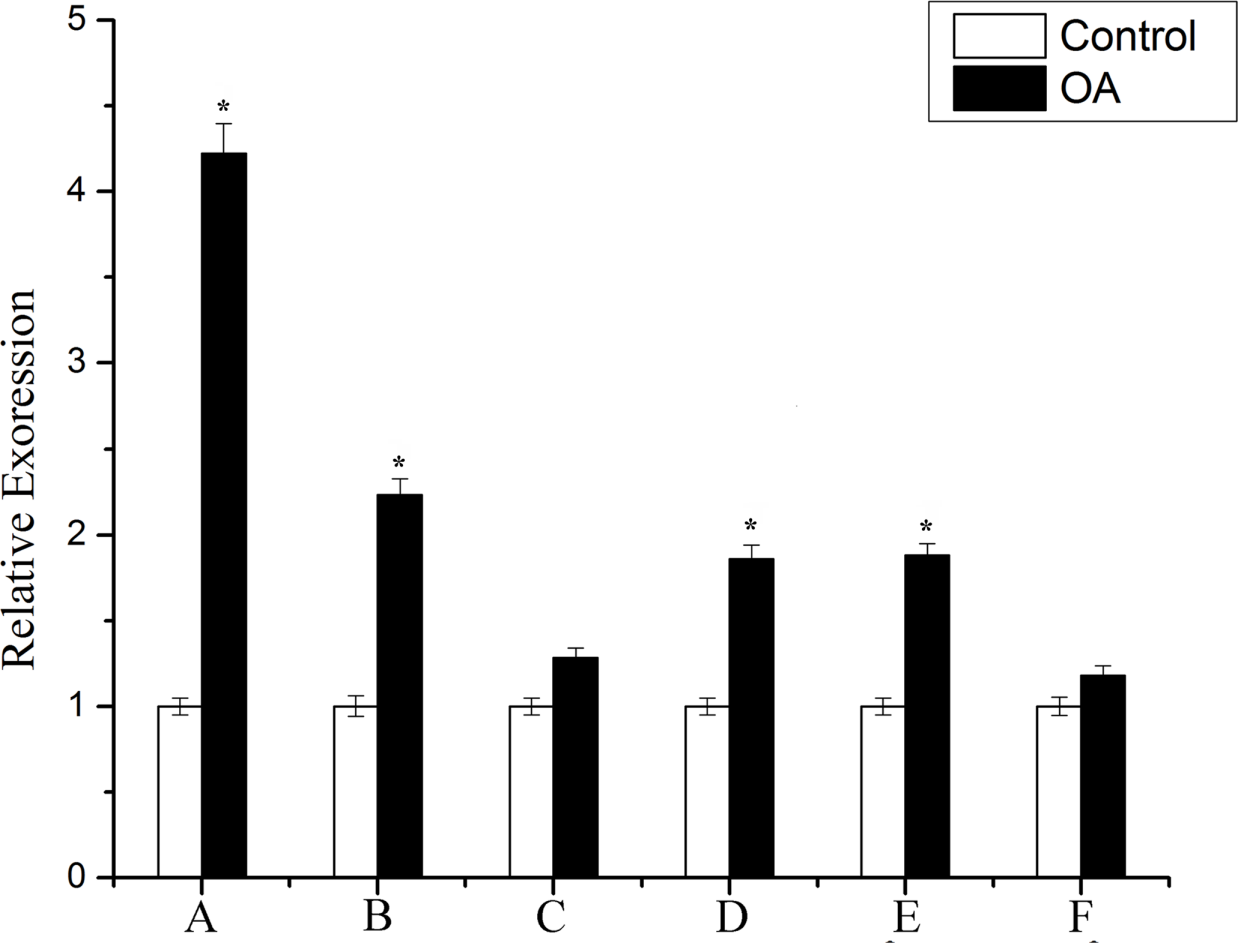
The transcriptional profile of topoisomerase genes in response to OA treatment. *top 1*,*topoisomerase I; Top 6*, *DNA topoisomerase 6; Top 2-like*, *DNA topoisomerase 2-like; Top 1*, *DNA topoisomerase I; pTop 1*, *putative DNA Topoisomerase I; Top2bp1*, *DNA topoisomerase 2-binding protein 1*. The y-axis indicates relative expression values and the x-axis indicates different genes. The relative gene expression levels were normalized to those of the Actin gene. All mRNAs in this experiment were extracted from seedlings treated for 3 d. Relative expression ratio of each sample is compared to the control group. Each experiment was repeated three times and the average value was shown with standard deviation. *P<0.05, as compared to the control group by t-test.

To determine the exact phase of the cell cycle arrested by Okadaic acid, mitotic phase was divided into two phases, phase I representing preprophase and phase II including from prophase to telophase (Fig 4A). Analysis of 1000 cells revealed that after treating with OA, cells in phase I were significantly increased and cells in phase II were decreased compared with the control group, proving that the cell cycle was exactly arrested at preprophase (Table 2; Fig 4B and 4C). Since the cell cycle can be delayed at G_2_-phase checkpoint due to DNA damage, TUNEL assays were performed. TUNEL assay is used for detecting DNA breaks. The treated group displayed the DNA damage signal in TUNEL compared with the control group after 3d of exposure, suggesting that genomic DNA did break in the cell cycle arrest (Fig 5). Combining these results with the chromosomes decondensation throughout M phase, we tested expression of topoisomerase genes, which plays a key role at the transition point from chromatin to chromosome, as well as the DNA repair. The results showed that expression of topoisomerase genes in treated groups was higher than that in the control group (Fig 6).

**Table 2:** Statistical data of phase analysis.

Phases	CK		OA	
	n	%	n	%
Interphase	889	88.9	870	87
Phase I	67	6.7	110	11
Phase II	44	4.4	20	2
∑	1000	100	1000	100

### Histone H4 lysine 5 acetylation is elevated in the DNA damage response after OA treatment

**Fig 7:**
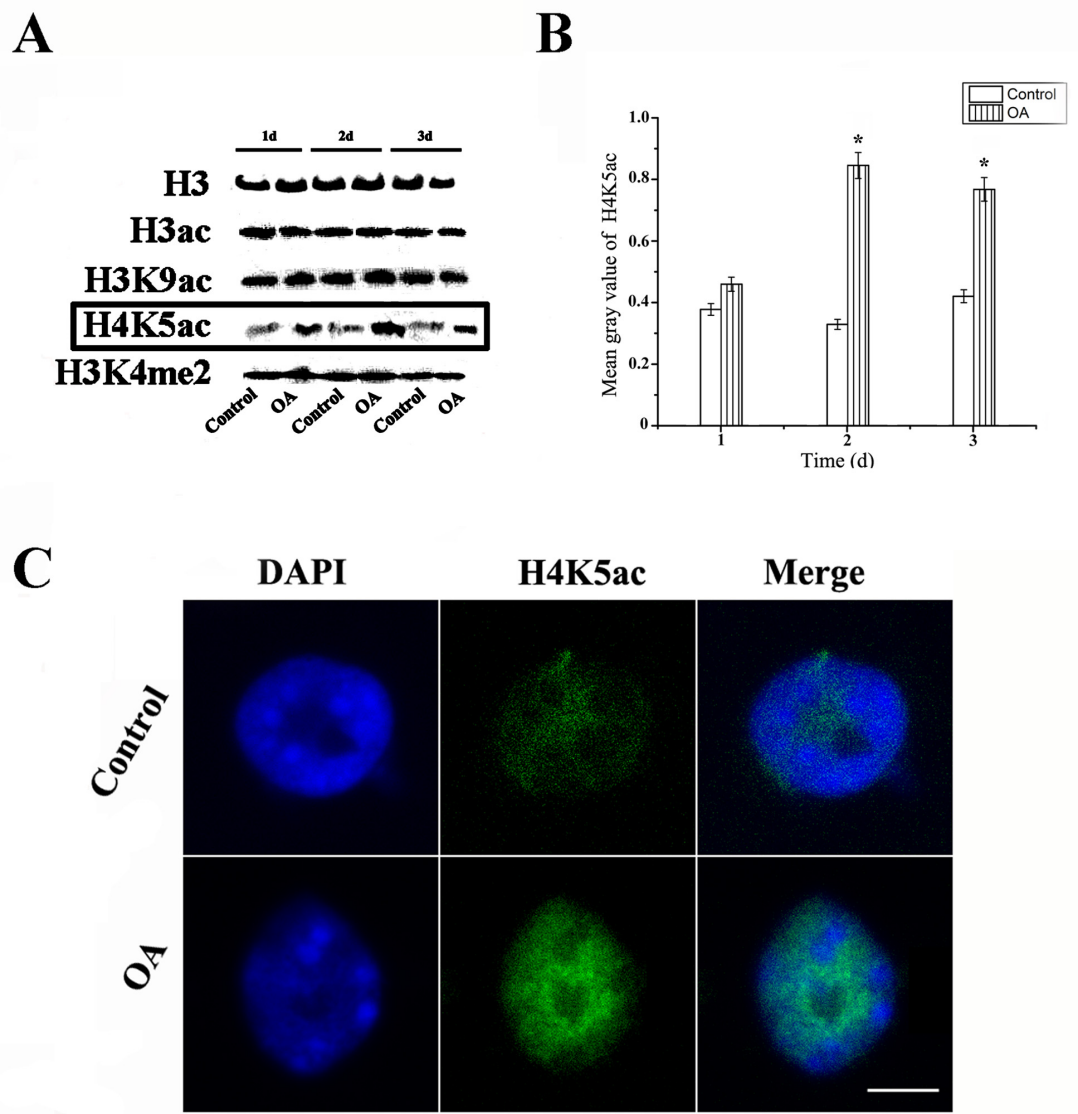
OA treatment leads to increase of H4K5ac. (A) The levels of H3ac, H3K9ac, H4K5ac and H3K4me2 were measured by Western blot assays. H3 was used as an internal control. (B) The histogram quantitatively displaying the mean gray values of H4K5ac that were increased by more than 100% after treatment with OA for 1–3 d. (C) Nuclei from seedlings untreated (Control) or treated with 100 nM OA for 3 d were subjected to immunofluorescence using antibodies against H4K5Ac and counterstained with DAPI (DAPI panel). The ‘Merge’ panel shows a merged image of blue and green staining. More than 200 nuclei were analyzed. Bar = 10 μm. *P<0.05, as compared to the control group by t-test.

It has been demonstrated that histone tail acetylation is required directly for DNA repair [16]. Therefore, western blotting assay was performed to investigate the state of various histone modifications during the arrest of cell cycle, using modification antibodies against H3ac, H3K9ac, H4K5ac, H3K4me2 and H3. Although most of histone modification levels kept stable, H4K5ac was significantly increased under OA treatment compared with the control group (Fig 7A and 7B), which agree with the reported result that H4 acetylation is interrelated with DNA repairs during cell cycle. Further immunostaining of nuclei also showed that H4K5ac signals were enhanced after treatment with OA (Fig 7C).

### ROS accumulation is involved in the arrest of cell cycles

**Fig 8:**
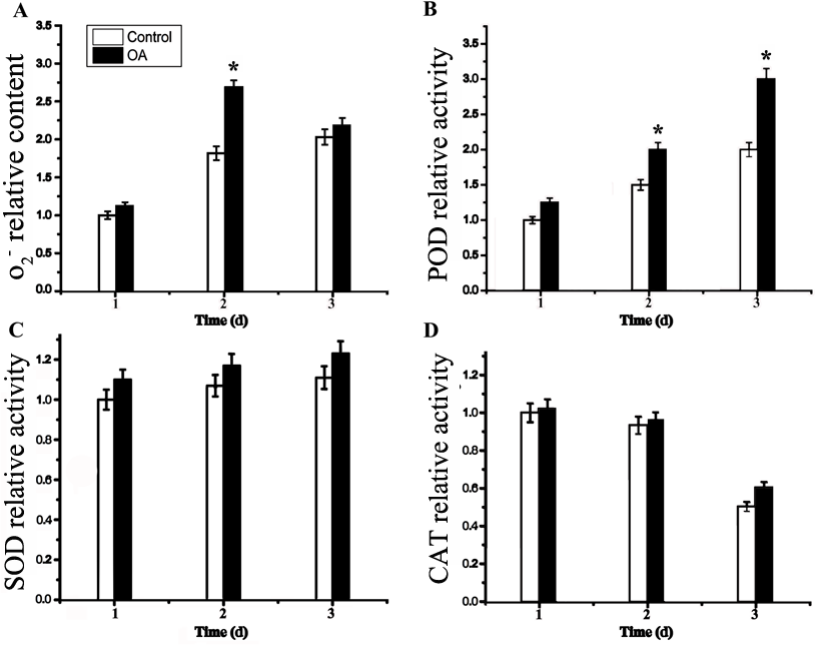
Variations of the O2- ion and ROS-related enzyme activities in response to OA treatment. (A) Concentration of negative oxygen ion (O_2_^-^). Activity of peroxidase (POD) (B), superoxide dismutase (SOD) (C), and catalase (CAT) (D). Relative concentration and enzyme activity of the control group (1 d) were defined as 1.0. Each assay was repeated three times for every sample from three independent experiments. The average value was shown with standard deviation. *P<0.05, as compared to the control group by t-test.

**Fig 9:**
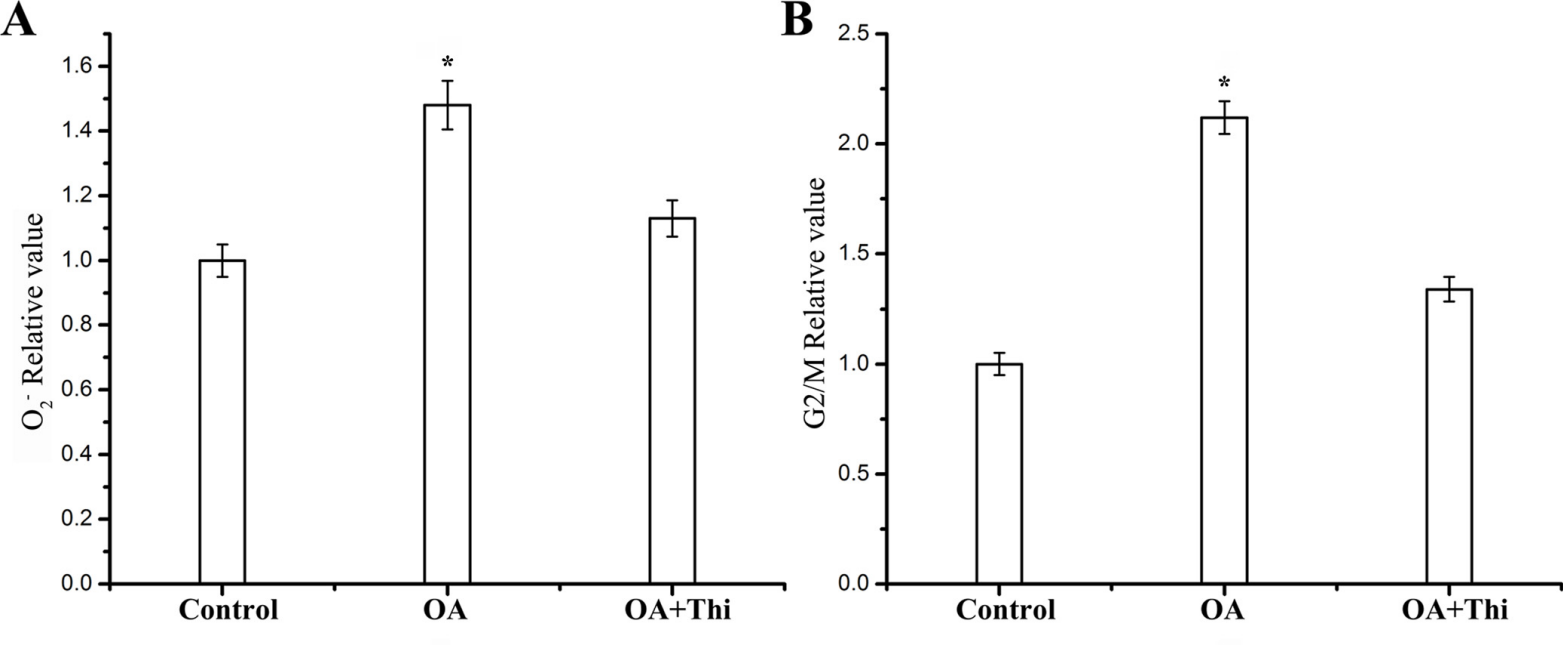
The cell cycle arrest caused by OA was released by antioxidant thiourea. (A) Concentration of negative oxygen ion (O_2_^-^) after treatment with OA or OA plus thiourea for 48 h. (B) The rate of cells in G_2_/M phase after treatment with OA or OA plus thiourea for 48 h. Relative O_2_^-^ concentration and G_2_/M value of the control group were both defined as 1.0. Each assay is repeated three times for every sample from three independent experiments and the average value was shown with standard deviation. *P<0.05, as compared to the control group by t-test.

Oxygen pressure is thought to be related with the cell cycle arrest via DNA damage. In order to verify whether ROS accumulation was involved in the preprophase arrest induced by OA, we measured the concentration of O_2_^-^ in seedling leaves. The results showed that the concentration of O_2_^-^ kept higher levels in the treated group than that in control group, suggesting the involvement of ROS in the preprophase arrest (Fig 8A). Furthermore, activities of ROS-related enzymes, catalase (CAT), superoxide dismutase (SOD) and peroxidase (POD), were found to be raised in treated groups compared with the control group (Fig 8B and 8D). To further confirm the involvement of ROS in the cell cycle arrest, antioxidant thiourea was tested for reducing concentration of O_2_^-^ molecule in leaf cells of treated groups. OA exposure for 2 d caused an increase of O_2_^-^ in leaves, and OA plus thiourea treatment did not cause significant changes in O_2_^-^ concentration (Fig 9A), suggesting that thiourea could decrease OA-induced ROS accumulation. Cell cycle analysis showed that additional oxygen stress induced the cell cycle arrest could be partly released by the addition of antioxidant thiourea (Fig 9B), proving that O_2_^-^ molecule played an important role in the preprophase arrests induced by OA.

## Discussion

OA was applied to investigate the relation between histone modification and the cell cycle regulation in maize seedlings in this study. H3S10 phosphorylation is a key histone modification in mitosis and starts in early prophase and sustains until telophase [10]. The dynamic process of H3S10ph in mitosis, associated with chromosome remodeling, is conserved during evolution in eukaryotes, which suggested this modification is very important for chromatin remolding. In this study the maize seedling growth was inhibited and cell cycle was arrested, accompanied with the dispersed signal distributions of H3S10ph on chromosomes after OA treatment. The results further provided evidence that the cell cycle was arrested at preprophase, the transition period from chromatin to chromosome. Treatment of tobacco cells with OA resulted in similar chromosome condensation abnormalities, and mitosis was arrested at G2-M transition [37]. Chromatin become condensed gradually in interphase and proprephase, and highly condensed chromosomes facilitate accurate segregation of the genetic material to daughter cells [38]. We speculated that OA treatment could affect chromatin structure by means of the change in H3S10 phosphorylation signal, consequently causing cell cycle arrest in maize seedlings.

It has been reported that histone modification inhibitors could increase ROS production [39, 40]. OA can both affect H3S10 phosphorylation and ROS/MAPK signaling pathways *in vitro* [41, 42]. The ROS mediated cell cycle arrest via DNA damage responses, and the DNA damage response signal-transduction pathway regulates cell cycle progression [43]. In the research of cell cycle alterations induced by PM fraction (PM2.5; aerodynamic diameter≤2.5 μm), ROS formation was reported to be involved in the cell cycle delay [44]. Similarly, our results revealed that OA induced ROS accumulation in the cell and the use of deoxidizer reduced the content of O_2_^-^ and relieved the arrest of cell cycle induced by OA, confirming that the ROS participated in the preprophase arrest. Furthermore, it has been reported that eukaryotic DNA topoisomerase participate in the final stage of chromosome condensation [45, 46]. In OA-treated maize seedlings, expression of DNA topoisomerase family all kept relatively higher levels than that in the control group, suggesting that the cells were undergoing a repairing process with the aim of releasing cell cycle from decondensation caused by DNA damage.

However, the packaging of DNA into chromatin is likely to be a barrier to access of DNA repair related complex. The previous works indicated that histone hyperacetylation is concerned with responses to DNA damage induced by ROS accumulation [47]. Histone acetylation is related to DNA repair [16, 48], because decondensation of the nucleosome structure mediated by histone acetylation renders chromatin more accessible to nuclear protein factors [49]. Histone acetylation can reduce the interactions between histones and DNA, thereby relaxing the DNA structure and easily recruiting DNA repair proteins for DNA repair [50]. So we speculated that the enrichment of H4K5ac after OA treatment is associated with the chromatin decondesation for efficient repair of DNA breaks through the genome, which consequently also cause preprophase arrest in maize.

This study demonstrates a mechanism of OA-induced alteration of cell cycle progression via augmented accumulation of ROS and the downstream DNA damage in the maize seedlings, and the changes in histone H3S10 phosphorylation and H4K5 acetylation signal are involved in this progression. The cell cycle is arrested at proprephase, the transition point from chromatin to chromosome, allowing more time for cells to detoxify ROS and to repair damaged DNA.

## Supporting Information

S1 Fig The expression level of *PP1* gene after OA treatment. The x-axis indicates the days after treatment with OA and the y-axis shows relative expression values. Expression values were normalized to those of the *beta actin* gene. The relative expression value of control group in 1d was assigned as 1. The experiments were repeated three times.*P<0.05, as compared to the control group by t-test.
(TIF)

## Abbreviations

OA: okadaic acid
ROS: reactive oxygen species
TUNEL: TdT-mediated dUTP nick end labeling
PP1: protein serine/threonine phosphatases-1
PP2: protein serine/threonine phosphatases-2
FITC: fluorescein isothiocyanate
DAPI 4': 6-diamidino-2-phenylindole
SOD: superoxide dismutase
POD: peroxidase
CAT: catalase activity detection
