# Supplementary figures and images for: Histone Modification Is Involved in Okadaic Acid (OA) Induced DNA Damage Response and G2-M Transition Arrest in Maize
Source: PLoS One. 2016 May 19;11(5):e0155852. doi: 10.1371/journal.pone.0155852 (PMC4873197; doi:10.1371/journal.pone.0155852)

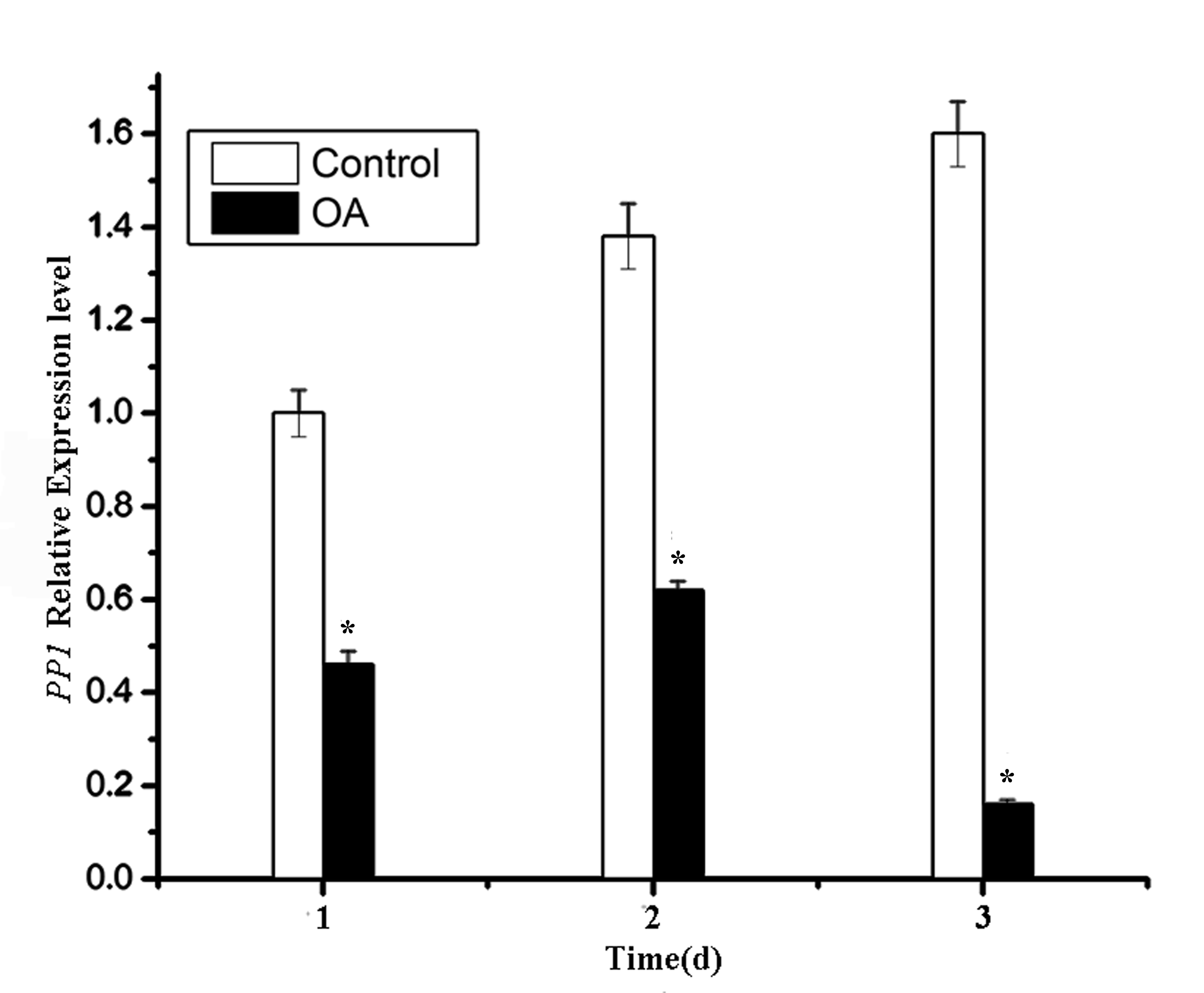

Supplement: S1 Fig — The x-axis indicates the days after treatment with OA and the y-axis shows relative expression values. Expression values were normalized to those of the beta actin gene. The relative expression value of control group in 1d was assigned as 1. The experiments were repeated three times.*P<0.05, as compared to the control group by t-test. (TIF) [file pone.0155852.s001.tif]
